# Comprehensive analysis of potential ceRNA network and immune cell infiltration in intervertebral disc degeneration

**DOI:** 10.1186/s13018-022-03331-x

**Published:** 2022-09-29

**Authors:** Xiaoyang Fang, Tian Tang, Daoxi Sun, Shuang Chen, Nan Wang, Lin Xie

**Affiliations:** Department of Spine Surgery, Affiliated Hospital of Integrated Traditional Chinese and Western Medicine, Nanjing University of Chinese Medicine, Nanjing, China

**Keywords:** Intervertebral disc degeneration, Competing endogenous RNA, Immune cell infiltration, Bioinformatics

## Abstract

**Background:**

Intervertebral disc degeneration (IDD) has become a serious public health problem, the mechanism of which is complex and still unclear. We aimed to construct a ceRNA network related to IDD to explore its pathogenesis.

**Methods:**

We downloaded the GSE67566, GSE63492, GSE116726 and GSE124272 datasets from GEO database, and obtained the differentially expressed RNAs. Then, we constructed a ceRNA network and the KEGG and GO enrichment analysis were performed. Finally, we performed immune cell infiltration analysis on the GSE124272 dataset and analysed the correlation between immune cell abundance and hub genes expression levels.

**Results:**

The ceRNA network included three down-regulated circRNAs: hsa_circ_0074817, hsa_circ_0002702, hsa_circ_0003600, three up-regulated miRNAs: hsa-miR-4741, hsa-miR-3158-5p, hsa-miR-508-5p, and 57 down-regulated mRNAs, including six hub genes: IGF1, CHEK1, CCNB1, OIP5, BIRC5, AR. GO and KEGG analysis revealed that the network is involved in various biological functions. Immune infiltration analysis showed that IDD was closely related to immune cell infiltration, and hub genes could further affect the development of IDD by affecting immune cell infiltration.

**Conclusion:**

This study identified the hsa_circ_0074817-hsa-miR-508-5p-IGF1/CHEK1/CCNB1, the hsa_circ_0003600-hsa-miR-4741-BIRC5/OIP5/AR and the hsa_circ_0002702-hsa-miR-3158-5p-IGF1/AR as important regulatory axis of IDD, which will help us gain further insight into the pathogenesis of IDD and determine potential therapeutic targets.

## Introduction

Lumbar degenerative disease (LDD) is a common disease in middle-aged and elderly people, with chronic low back pain as the main clinical manifestation. With the aging population, LDD has become a serious public health problem [1]. Intervertebral disc degeneration (IDD) is the pathological basis for its development [2], and a series of diseases such as lumbar disc herniation, endplate inflammation and lumbar spinal stenosis can develop as a result. IDD can occur in adolescence and gets progressively worse with age, with the prevalence of IDD exceeding 90% in people older than 50 years [3]. At present, the pathogenesis of IDD is still not specifically elucidated. Anti-inflammatory and analgesic drugs are often used in conservative treatment to relieve symptoms, and surgical treatment may still leave residual back and leg pain or generate new pain due to degeneration of adjacent segments. Therefore, it is helpful to understand the pathogenesis and find key targets for the treatment of IDD.

With the continuous development of biology, microRNAs (miRNAs), circular RNAs (circRNAs), and long non-coding RNAs (lncRNAs) have been shown to be involved in the occurrence and development of IDD and are considered to be the key to diagnosis and treatment. MiRNAs can bind to target mRNAs to destabilize them and inhibit mRNAs translation or cause their degradation, thus achieving post-transcriptional regulation of gene expression. MiRNAs play an important role in the biological process of IDD and participate in it by regulating inflammatory response, cell proliferation, cell apoptosis and other pathways [4, 5]. Dong found that miR-640 inhibited the expression of β-catenin and EP300, thereby suppressing WNT signalling and inducing nucleus pulposus cell degeneration [6]. Based on this, they proposed that miR-640 inhibitor therapy is a potential therapeutic target for low back pain. As research progressed, circRNAs were found to regulate gene expression by sponging miRNAs to achieve the purpose of regulating diseases [7]. Nowadays, more and more studies have shown that circRNAs also play an important role in IDD, participating in extracellular matrix metabolism, cellular senescence, apoptosis, inflammatory response and other processes [8]. Hu found that circRNA_0022382 ameliorated IDD by sponging miR-4726-5p to downregulate TGF-β3 expression, suggesting that circRNA_0022382 may serve as a new way to prevent and treat IDD [9]. The microenvironment of intervertebral disc also has an important impact on the development of IDD. Some miRNAs (miR-299-5p、miR-17), mRNAs (TGF-β、IL-1β) and lncRNA (SNHG5) have been reported to affect the development of IDD by mediating immune cells [10, 11]. But so far, no research has thoroughly clarified the pathogenesis of IDD and the ceRNA regulatory network of circRNAs remains to be explored. Therefore, this study will mine the microarray datasets in the GEO database to find differentially expressed RNAs and construct a circRNA-miRNA-mRNA ceRNA network to explore the pathogenesis of IDD and seek new targets with the aim of providing new options for treatment.

## Materials and methods

### Data source

The GEO database (https://www.ncbi.nlm.nih.gov/geo/) is an international public database containing multiple gene expression datasets. We obtained four IDD microarray datasets (circRNA microarray set, GSE67566; miRNA microarray sets, GSE63492 and GSE116726; mRNA microarray set, GSE124272), and the specific information is shown in Table 1. Five IDD samples of nucleus pulposus derived from patients with IDD and five control samples derived from cadaveric discs were included in the GSE67566 and GSE63492 datasets. Three IDD samples of nucleus pulposus derived from patients with IDD and three control samples derived from fresh traumatic lumbar fracture patients were included in the GSE116726 dataset. Eight IDD samples from patients with lumbar disc prolapse and eight control samples were included in the GSE124272 dataset.

**Table 1:** IDD datasets included for analysis

Datasets	Platform information	Control	IDD
GSE67566	GPL19978 Agilent-069978 Arraystar Human CircRNA microarray V1	5	5
GSE63492	GPL19449 Exiqon miRCURY LNA microRNA Array, seventh generation REV—hsa, mmu & rno (miRBase v18.0)	5	5
GSE116726	GPL20712 Agilent-070156 Human miRNA [miRNA version]	3	3
GSE124272	GPL21185 Agilent-072363 SurePrint G3 Human GE v3 8 × 60 K Microarray 039494 [Probe Name Version]	8	8

### Identification of differentially expressed RNA

Using the R limma package to convert probe IDs and setting “*p* value < 0.05 and |logFC|> 1” to obtain differentially expressed circRNAs(DEcircRNAs)、differentially expressed miRNAs(DEmiRNAs) and differentially expressed mRNAs(DEmRNAs). Heatmap and volcano map were plotted with R package heatmap and ggplot2, respectively.

### Prediction of miRNA target genes

In order to establish the relationship between miRNA and circRNA, we used the circBanK database to predict the circRNAs of DEmiRNAs which were obtained by intersecting GSE63492 and GSE116726.The conservative circRNAs with coding_prob ≥ 0.9 were selected as the prediction results. In order to establish the relationship between miRNA and mRNA, we used the TargetScan database to predict relevant mRNAs and discarded mRNAs with cumulative weighted context ++ score of 0.

### Construction of ceRNA network and identification of hub genes

The miRNA-circRNA relationship pairs were constructed by intersecting the predicted circRNAs and DEcircRNAs, and the miRNA-mRNA relationship pairs were constructed by intersecting the predicted mRNAs and DEmRNAs. Subsequently, these relationship pairs were combined and presented by Cytocsape3.8.0. Then, eight common algorithms (degree, eccentricity, MNC, closeness, radiance, stress, betweenness, and dmnc) in the CytoHubba plug-in were used to screen hub genes. The top 15 results were intersected to obtain the final hub genes.

### Go and KEGG enrichment analysis

The Go function and KEGG pathway enrichment analysis of mRNA were performed through "clusterprofiler" in R software Bioconductor, and screening was carried out under the conditions of *p* value < 0.05 and *q* value < 0.05. Finally, we used ggplot2 package to present the results.

### Immune infiltration analysis

Based on the CIBERSORT algorithm, the abundance of 22 types of immune cells in IDD and healthy tissues in GSE124272 dataset were detected, and the infiltration of these 22 types of immune cells was compared. In addition, we also analysed the correlation between hub genes in ceRNA network and 22 immune cells. Pearson correlation analysis was used for data in-line with normal distribution, otherwise Spearman correlation analysis was used.

## Results

### Differentially expressed RNA

**Fig. 1:**
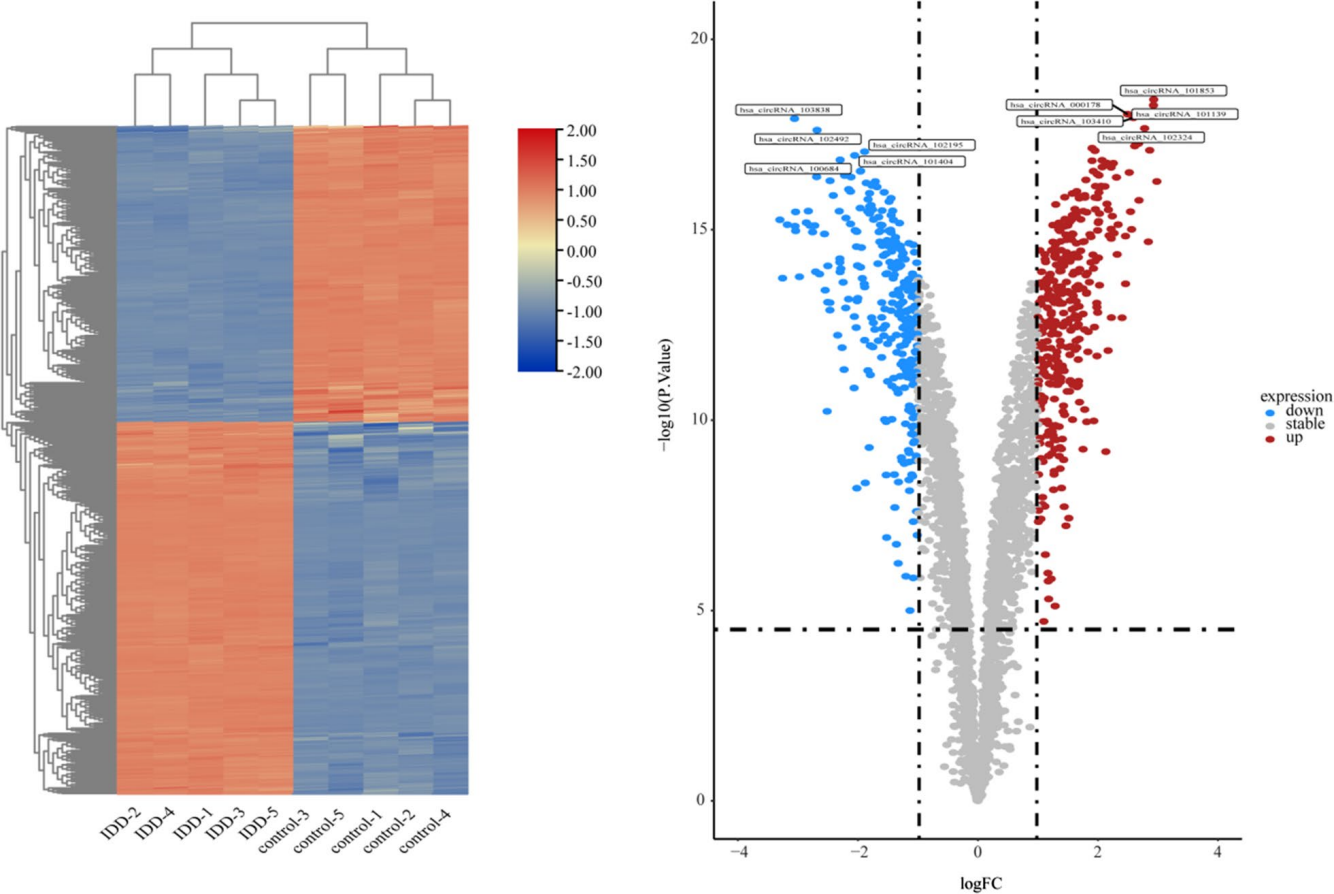
Results of DEcircRNAs analysis of GSE67566. The left: Heatmap of up/down-regulated circRNAs in IDD. The right: Volcano map of up/down-regulated circRNAs in IDD. The red dots represent up-regulated circRNAs and the blue dots represent down-regulated circRNAs

**Fig. 2:**
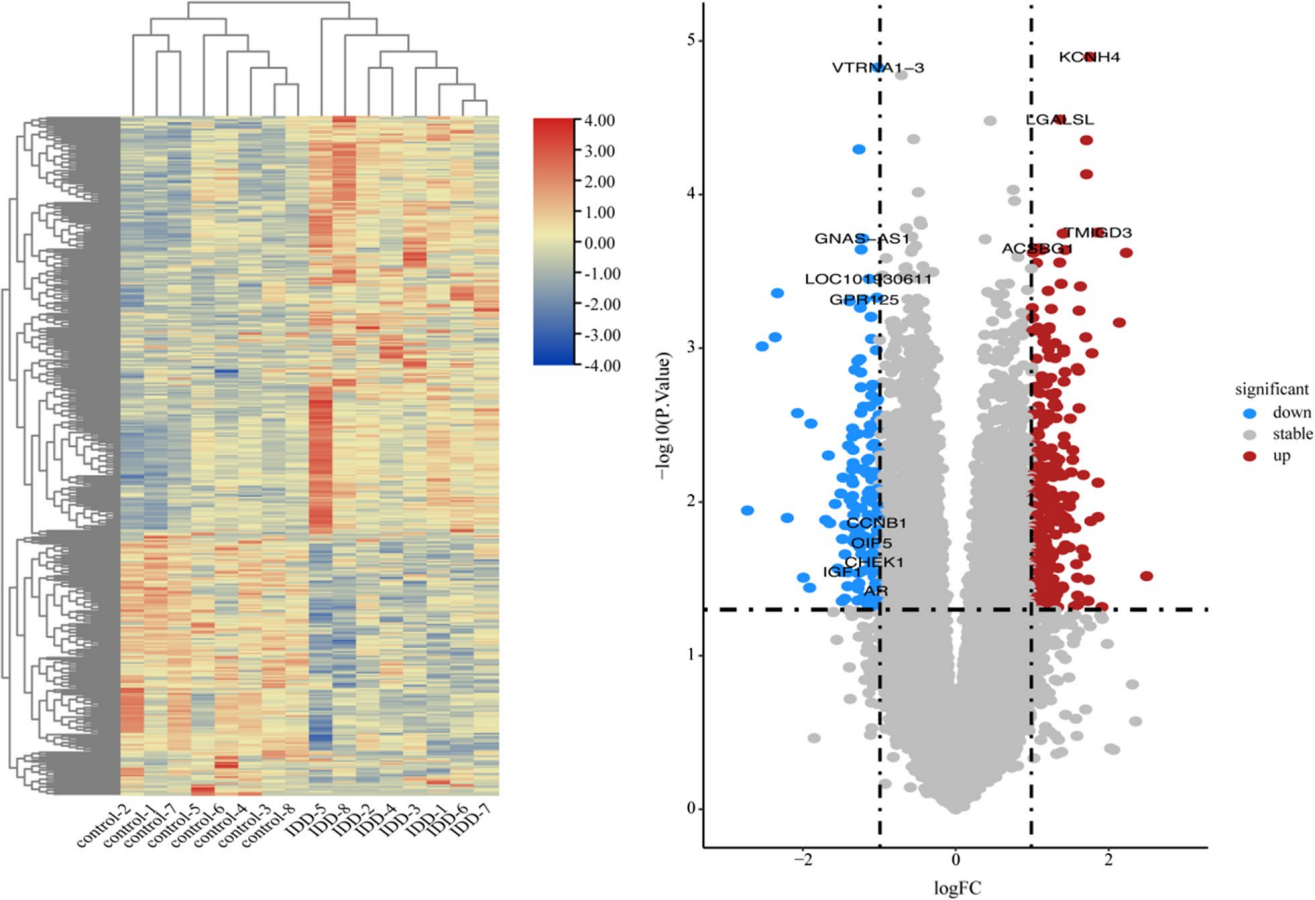
Results of DEmRNAs analysis of GSE124272. The left: Heatmap of up/down-regulated mRNAs in IDD. The right: Volcano map of up/down-regulated mRNAs in IDD. The red dots represent up-regulated mRNAs and the blue dots represent down-regulated mRNAs

**Fig. 3:**
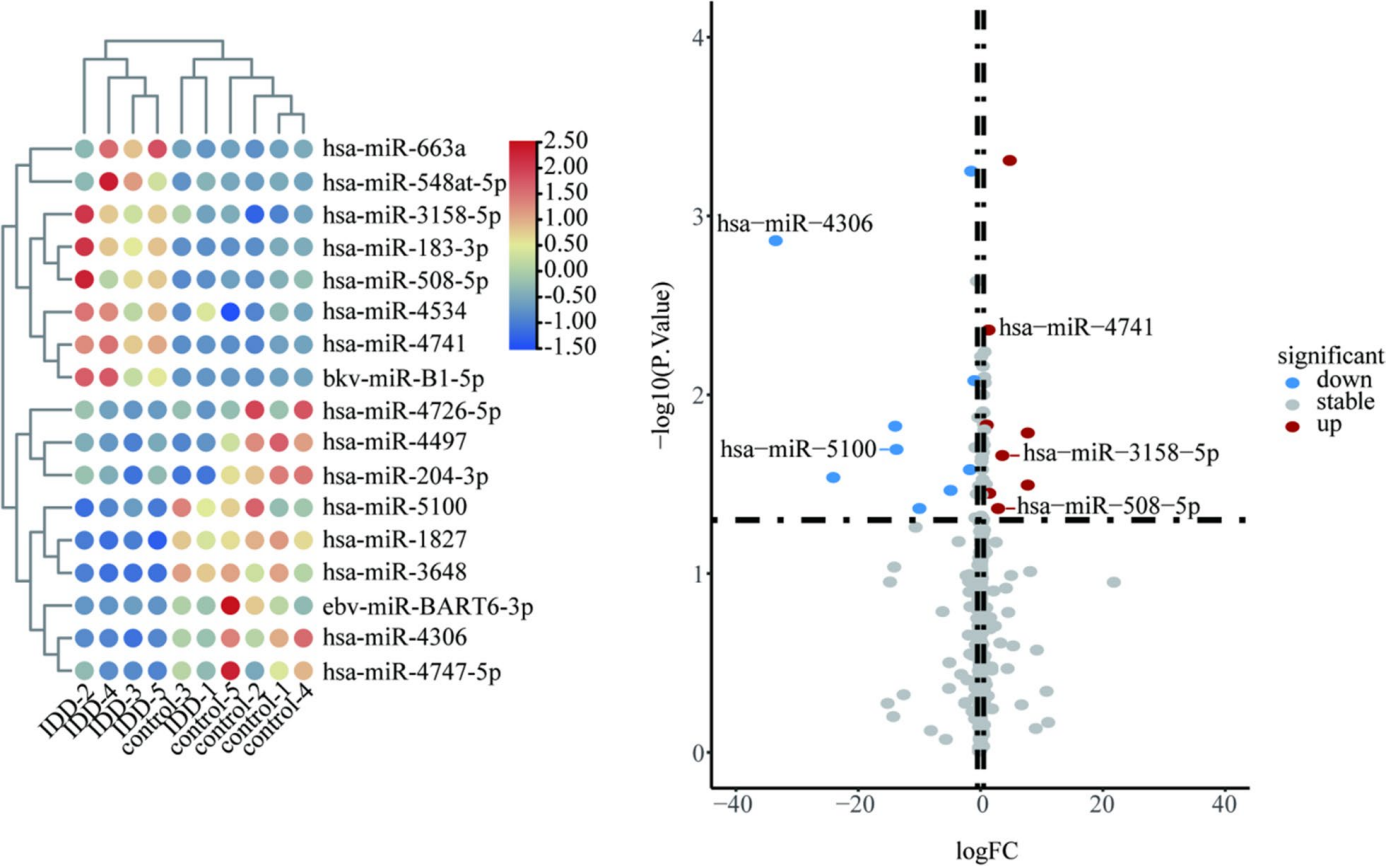
Results of DEmiRNAs analysis of GSE63492. The left: Heatmap of up/down-regulated miRNAs in IDD. The right: Volcano map of up/down-regulated miRNAs in IDD. The red dots represent up-regulated miRNAs and the blue dots represent down-regulated miRNAs

**Fig. 4:**
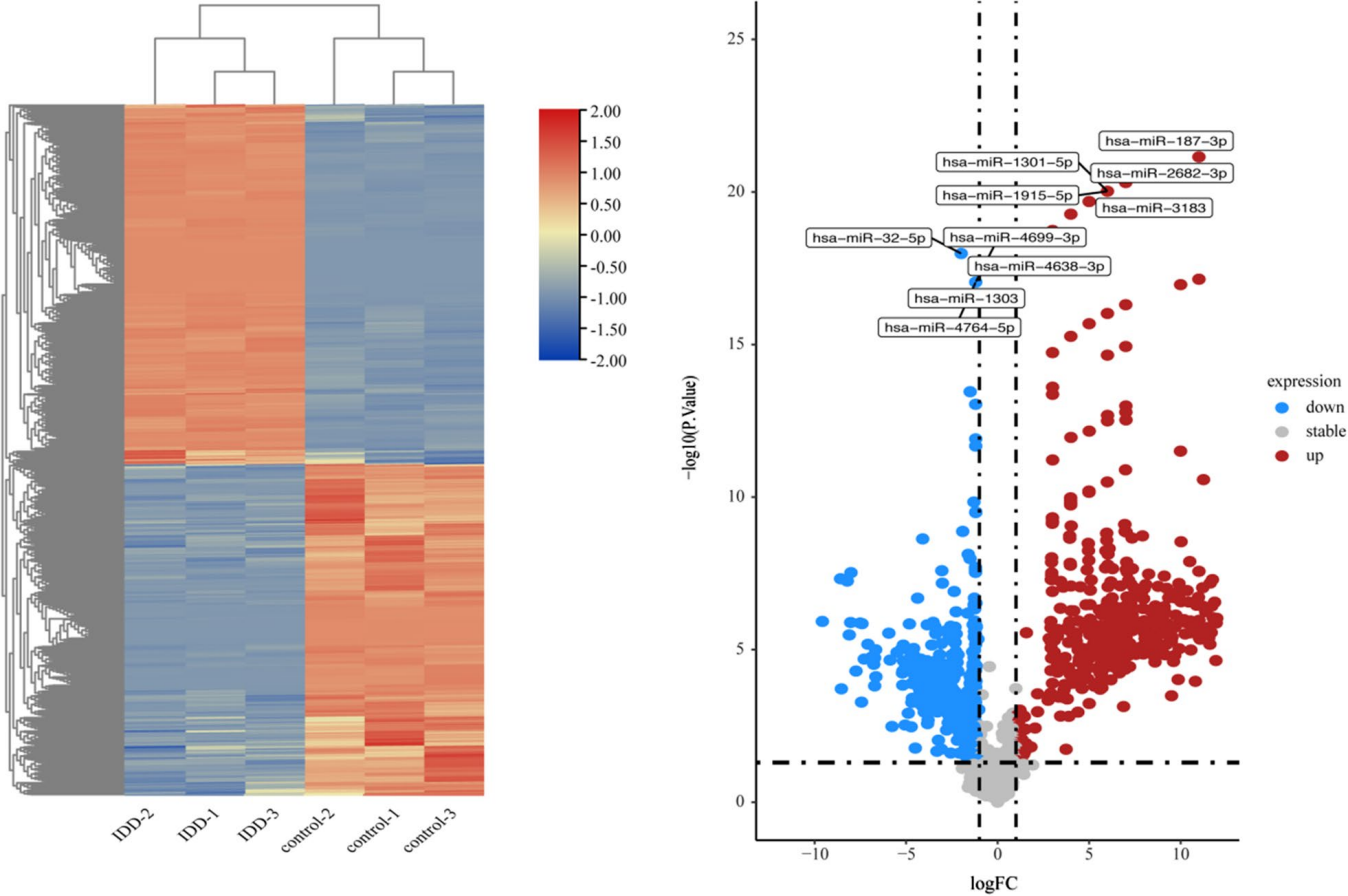
Results of DEmiRNAs analysis of GSE116726. The left: Heatmap of up/down-regulated miRNAs in IDD. The right: Volcano map of up/down-regulated miRNAs in IDD. The red dots represent up-regulated miRNAs and the blue dots represent down-regulated miRNAs

**Fig. 5:**
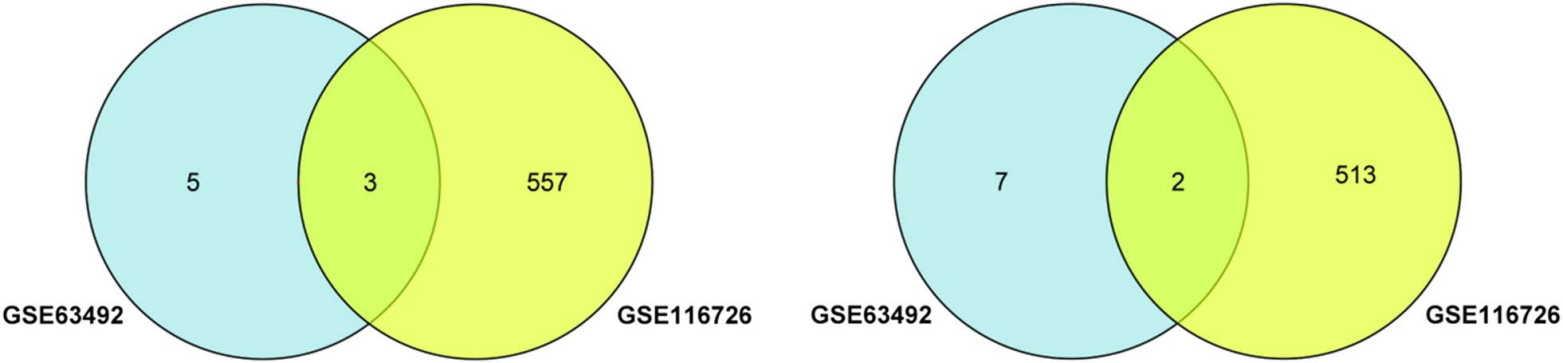
Venn diagram between GSE63492 and GSE116726. The left: up-regulated DEmiRNAs; the right: down-regulated DEmiRNAs

GSE67566 obtained 354 up-regulated DEcircRNAs and 282 down-regulated DEcircRNAs (Fig. 1). GSE124272 obtained 270 up-regulated DEmRNAs and 152 down-regulated DEmRNAs (Fig. 2). GSE63492 obtained 8 up-regulated DEmiRNAs and 9 down-regulated DEmiRNAs (Fig. 3), and GSE116726 obtained 560 up-regulated DEmiRNAs and 515 down-regulated DEmiRNAs (Fig. 4). After the intersection of these two datasets, three up-regulated DEmiRNAs (hsa-mir-4741, hsa-mir-3158-5p, hsa-mir-508-5p) and two down-regulated DEmiRNAs (hsa-mir-4306, hsa-mir-5100) were obtained (Fig. 5).

### Construction of miRNA relationship pairs

**Fig. 6:**
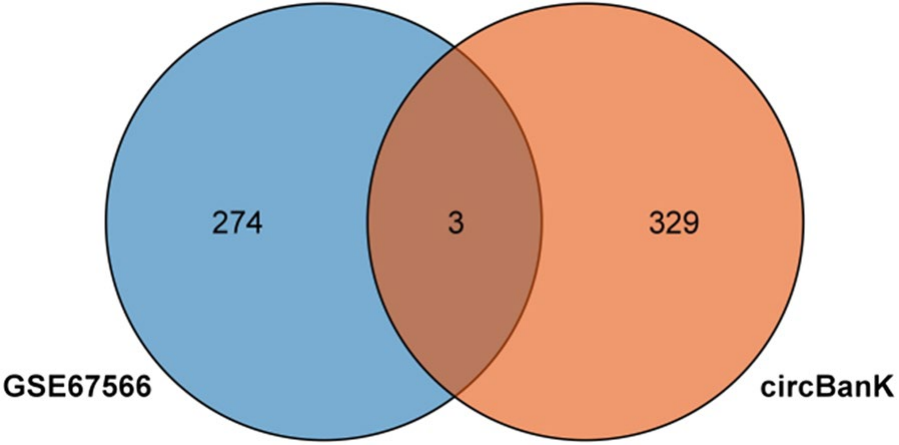
Venn diagram between down-DEcircRNAs and circBank results

**Fig. 7:**
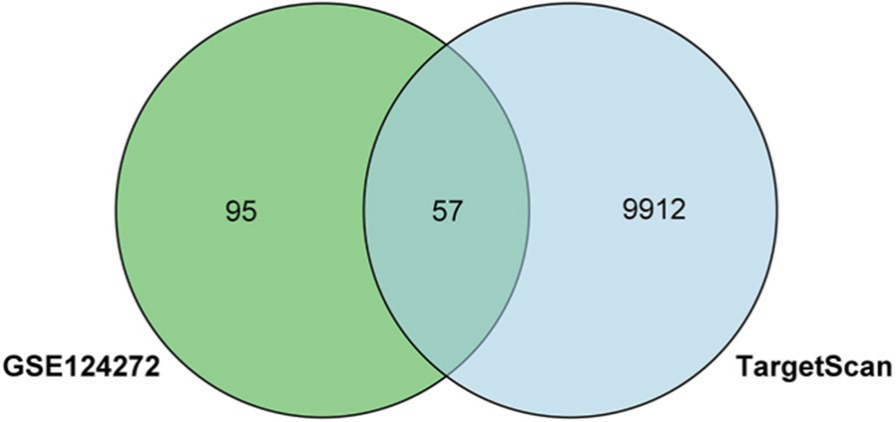
Venn diagram between down-DEmRNAs and TargetScan results

In order to construct the relationship pairs between miRNAs and circRNAs, the circBanK database was used to predict the circRNAs of intersecting DEmiRNAs. Among them, hsa-miR-4741, hsa-miR-3158-5p, and hsa-miR-508-5p predicted 104, 118, and 120 circRNAs, respectively, which were intersected with down-regulated DEcircRNAs, and finally three upstream circRNAs (hsa_circ_0074817, hsa_circ_0002702, hsa_circ_0003600) were obtained (Fig. 6). Hsa-miR-4306 and hsa-miR-5100 predicted 46 and 50 circRNAs, respectively, which had no intersection with up-regulated DEcircRNAs. The TargetScan database was used to predict the mRNAs of intersecting DEmiRNAs, of which 9969 mRNAs were predicted for up-regulated DEmiRNAs, and 57 downstream mRNAs were obtained by intersecting with down-regulated DEmRNAs (Fig. 7).

### Visualization of ceRNA network and identification of hub genes

**Fig. 8:**
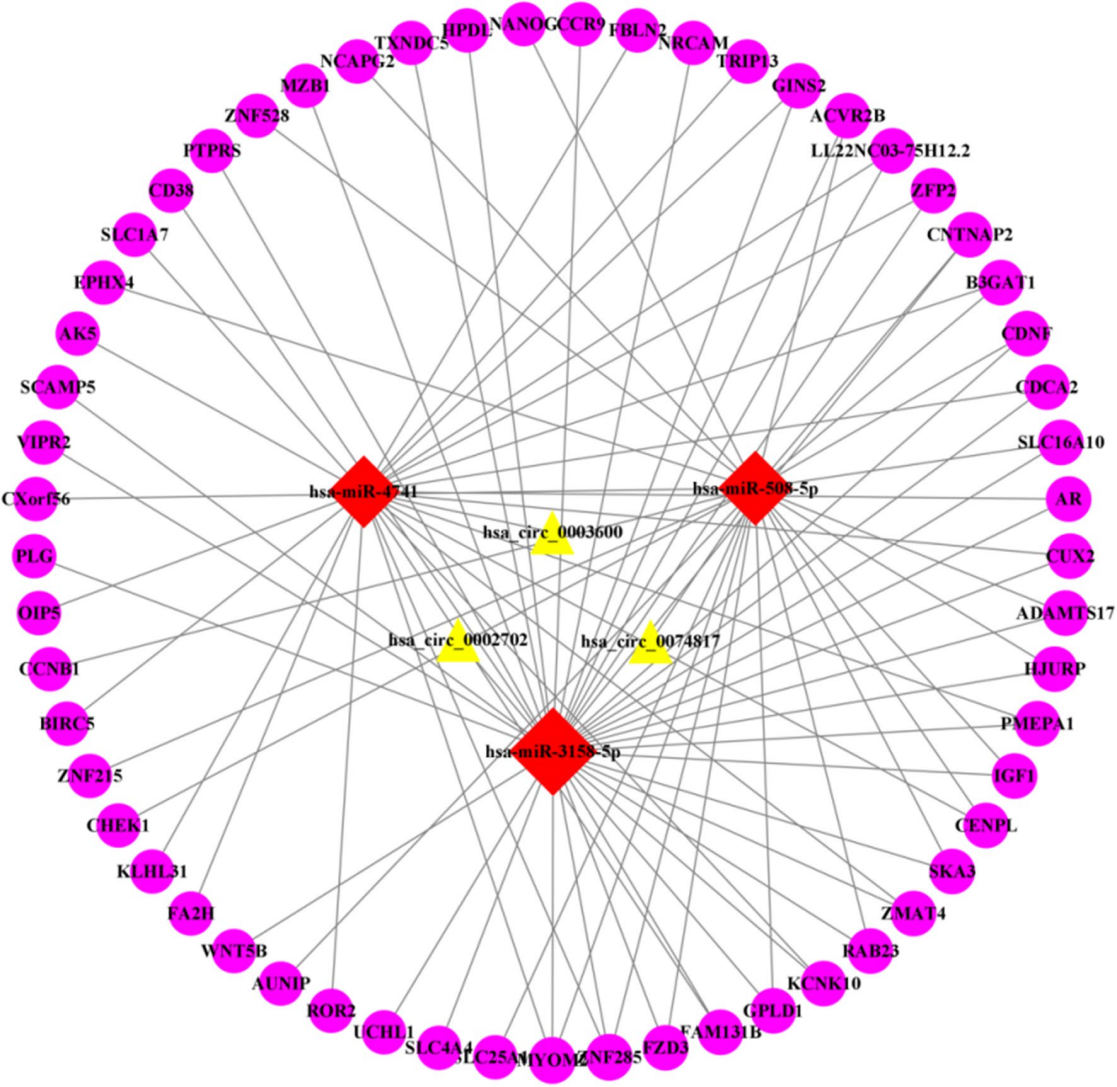
The ceRNA network of IDD. Red diamonds represent up-regulated miRNAs, yellow triangles represent down-regulated circRNAs, and pink circles represent down-regulated mRNAs

**Fig. 9:**
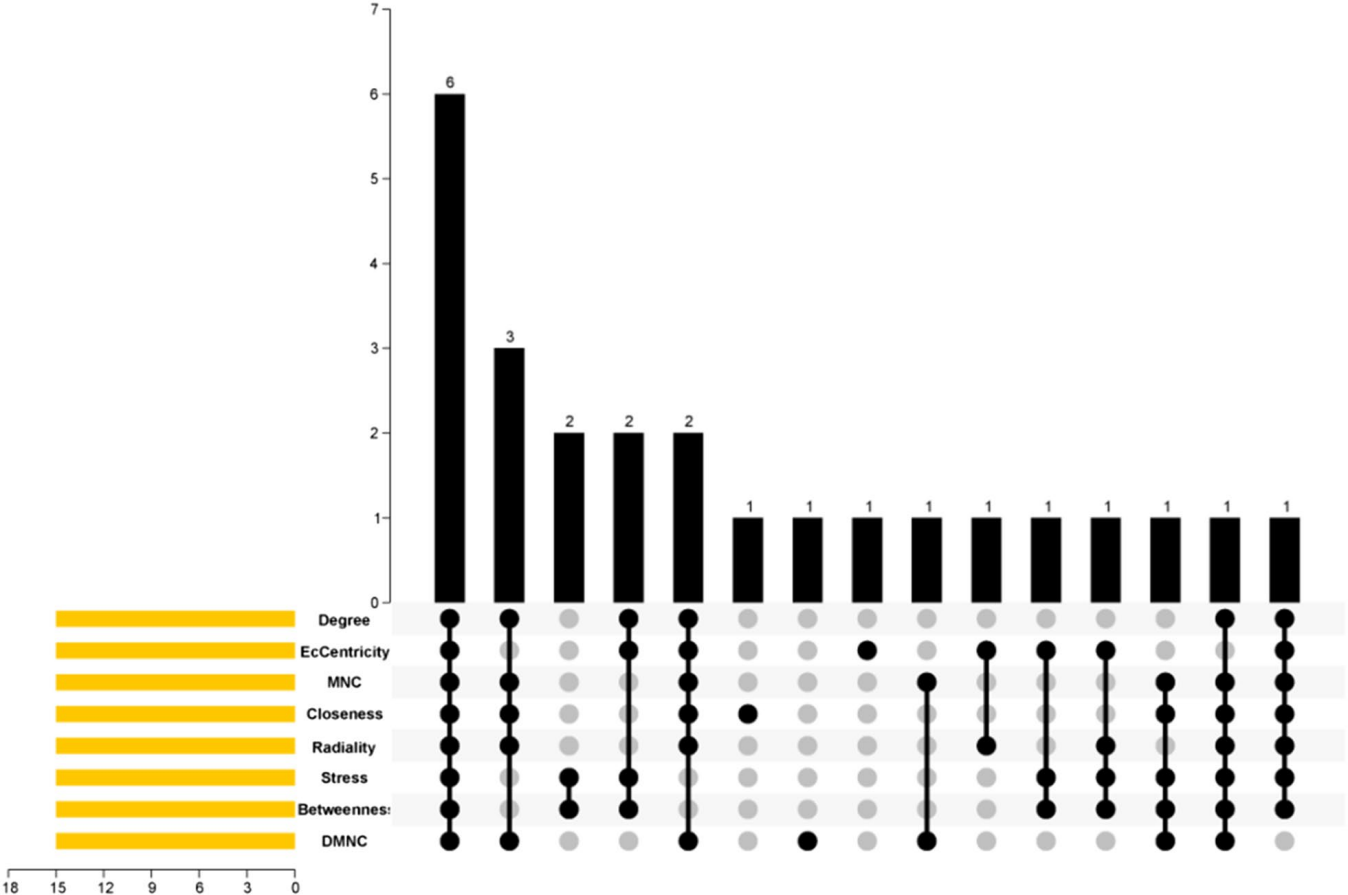
Venn diagram of hub genes

The miRNA relationship pairs obtained from the above predictions were combined to construct a circRNA-miRNA-mRNA ceRNA network, which was visualized according to the order of degree values with the help of Cytocsape software (Fig. 8). The hub genes were screened by CytoHubba plug-in, and the intersection was finally performed to obtain six mRNAs: IGF1, CHEK1, CCNB1, OIP5, BIRC5, and AR (Fig. 9).

### Go and KEGG enrichment analysis

**Fig. 10:**
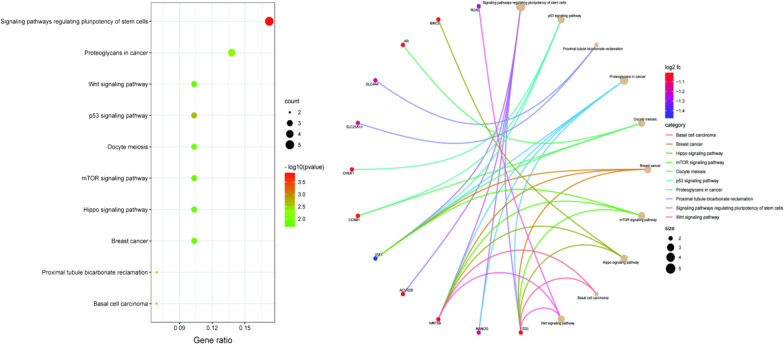
KEGG enrichment analysis of DEmRNAs. The left: bubble diagram of the top ten KEGG signalling pathways. Signalling pathways regulating pluripotency of stem cells and p53 signalling pathways were the most significantly enriched. The right: circle diagram of DEmRNAs specifically enriched in the signalling pathways. The enrichment of AR, CHEK1, CCNB1, BIRC5, ACVR2B, WNT5B and FZD3 was particularly significant

**Fig. 11:**
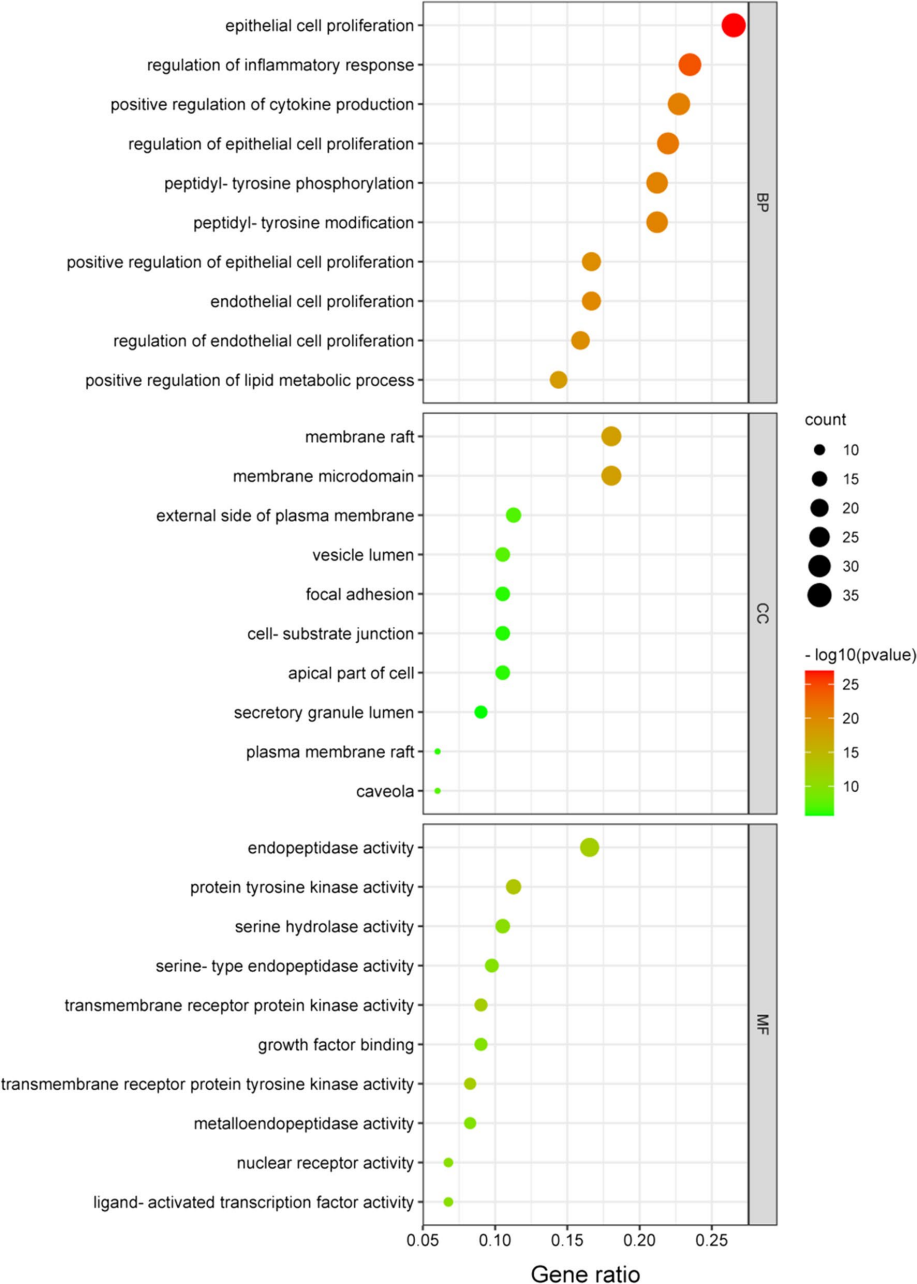
GO enrichment analysis of DEmRNAs

KEGG revealed 12 significant pathways (Fig. 10), of which signalling pathways regulating pluripotency of stem cells and p53 signalling pathways were the most significantly enriched, both related to IDD. GO analysis yielded 1929 biological process items, 59 cell composition items, and 130 molecular function items (Fig. 11). Among them, regulation of inflammatory response, positive regulation of cytokine production and endothelial cell proliferation involved in biological processes, cell-substrate junction involved in cell composition, and protein tyrosine kinase activity, ligand-activated transcription factor activity and growth factor binding involved in molecular functions are closely related to IDD.

### Immune cell infiltration in IDD and healthy samples

**Fig. 12:**
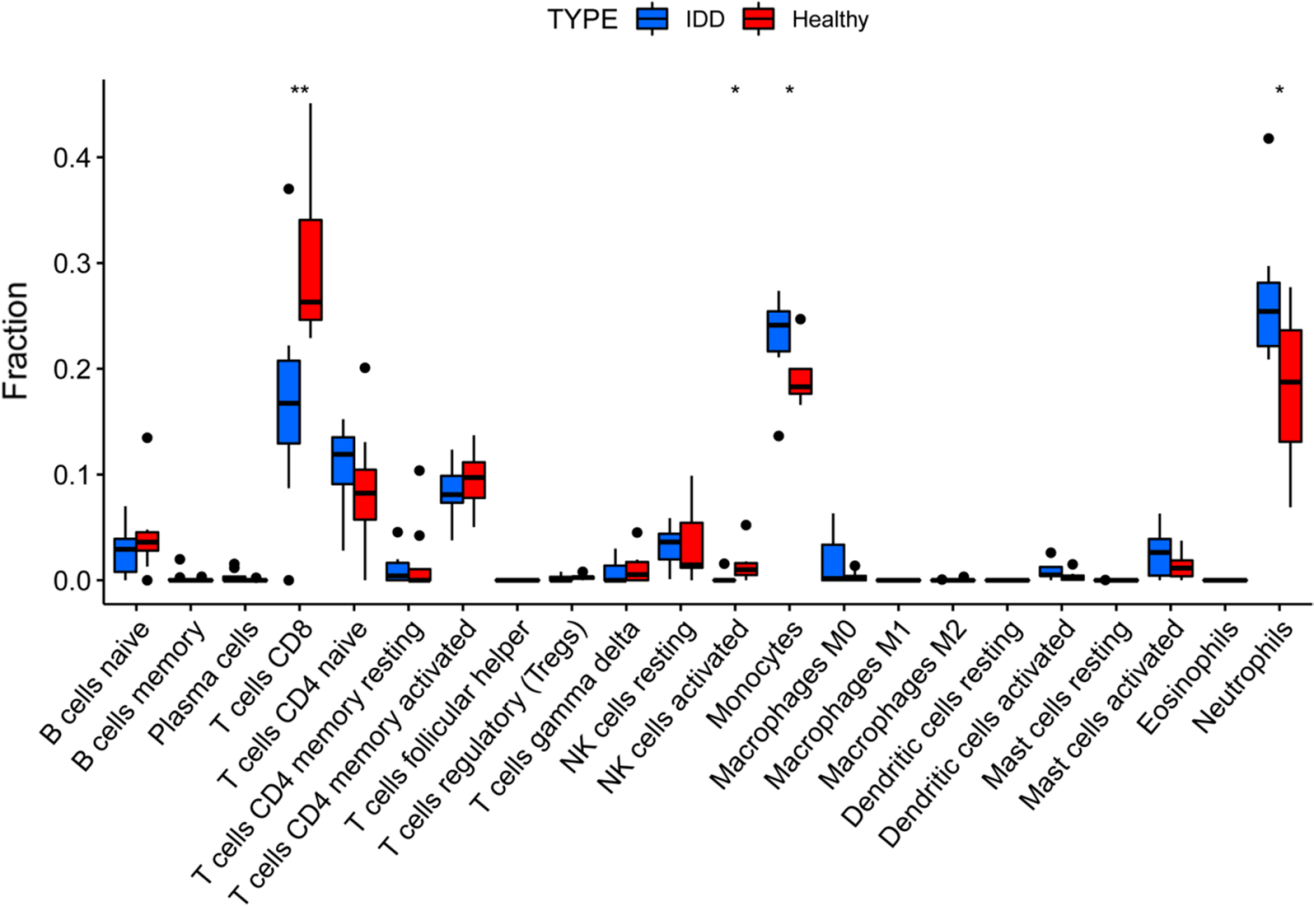
Abundance of 22 types of immune cell infiltrates in IDD and healthy samples. Compared with healthy samples, T cells CD8 and NK cells activated are low expressed in IDD, while monocytes and neutrophils are highly expressed. *P < 0.05, **P < 0.005

Boxplot showed that there are a large number of immune cells in healthy and degenerative intervertebral discs (Fig. 12). Compared with healthy intervertebral discs, T cells CD8 and NK cells activated are low expressed in IDD, while monocytes and neutrophils are highly expressed.

### Correlation analysis between expression levels of hub genes and immune cells

**Fig. 13:**
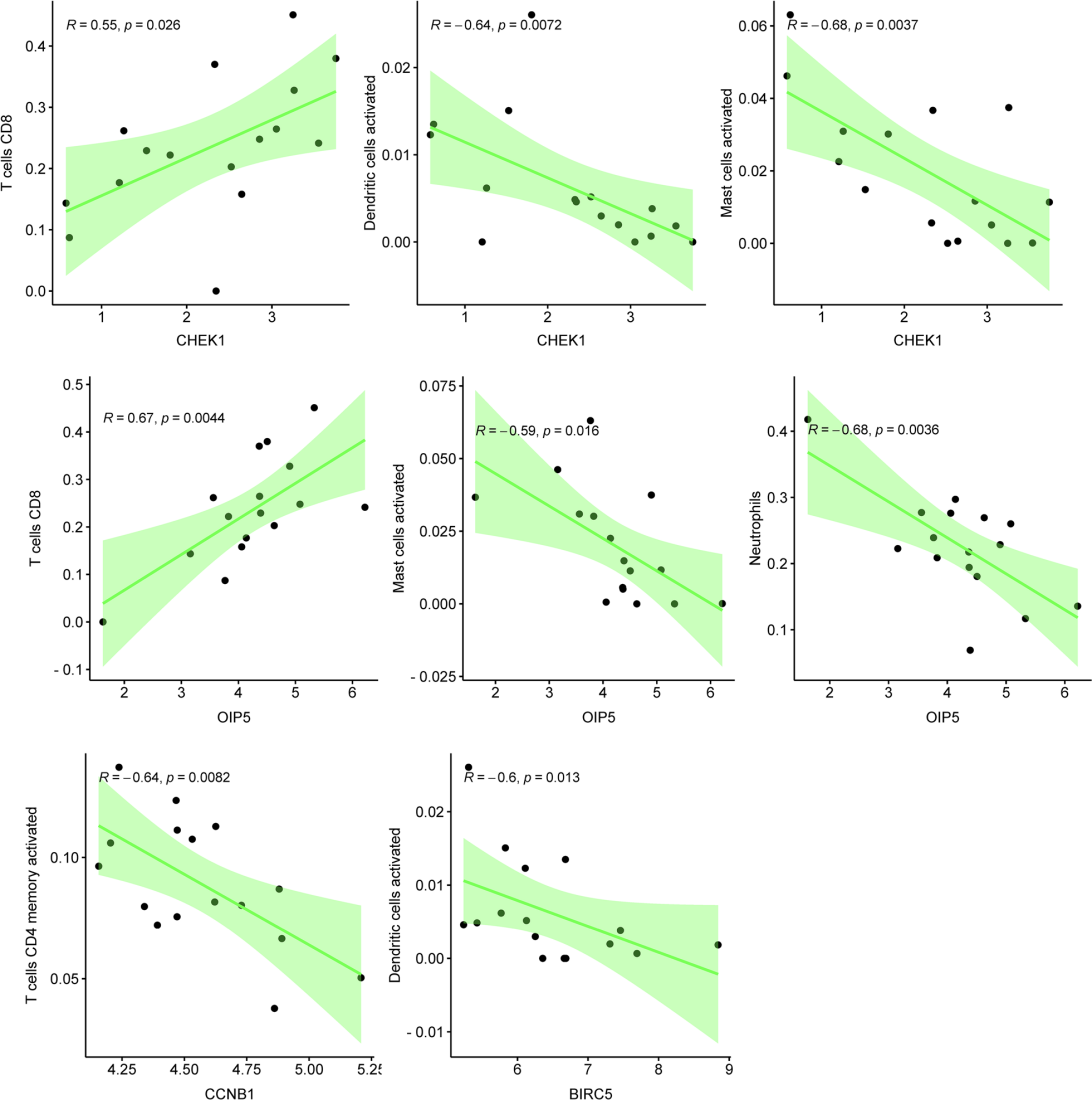
Correlation analysis between expression levels of hub genes and immune cells. CHEK1 is positively correlated with T cells CD8, but negatively correlated with dendritic cells activated and mast cells activated. OIP5 is positively correlated with T cells CD8, but negatively correlated with mast cells activated and neutrophils. CCNB1 is negatively correlated with T cells CD4 memory activated and the relationship between BIRC5 and dendritic cells activated is the same as before

The correlation analysis results (Fig. 13) showed that the high expression of CHEK1 leads to the high infiltration of T cells CD8, but the low infiltration of dendritic cells activated and mast cells activated. Similarly, the high expression of OIP5 leads to the high infiltration of T cells CD8, but the low infiltration of mast cells activated and neutrophils. The high expression of CCNB1 leads to the low infiltration of T cells CD4 memory activated and the relationship between BIRC5 and dendritic cells activated is the same as before.

## Discussion

IDD is the pathological basis of a series of spinal degenerative diseases and the main cause of chronic low back and leg pain. Its pathological features are the degeneration of the cartilage endplate and the fibrosis of the nucleus pulposus, resulting in the decline of nutrients and cell viability in the intervertebral disc [12]. The pathological process of IDD involves multiple risk factors, including genetics, aging, autoimmune response, mechanical load, smoking, diabetes and so on, but the exact pathogenesis remains unclear. With the development of bioinformatics, more and more studies use this method to explore the pathogenesis of diseases, and provide reliable research directions and treatment suggestions for clinic. The ceRNA network constructed in this study included three down-regulated circRNAs(hsa_circ_0074817, hsa_circ_0002702, hsa_circ_0003600), three up-regulated miRNAs(hsa-miR-4741, hsa-miR-3158-5p, hsa-miR-508-5p) and 57 down-regulated mRNAs, of which six hub genes(IGF1, CHEK1, CCNB1, OIP5, BIRC5, AR) were identified by CytoHubba.

GO enrichment analysis showed that IDD involved multiple organisms, cells, molecules and was closely related to the components, binding modes and functions of cells. Biological processes showed that IDD was closely bound up with regulation of inflammatory response, positive regulation of cytokine production, endogenous cell promotion and other items. A number of current studies have shown that various genes can regulate the proliferation and apoptosis of intervertebral disc cells, or promote and inhibit the release of inflammatory factors to regulate the development of IDD [4]. The cell composition involved cell substrate junction. In intervertebral discs, extracellular matrix is the most important link, and the changes of its biochemical components directly lead to IDD [13]. Protein tyrosine kinase activity, ligand activated transcription factor activity and growth factor binding in molecular function were closely related to IDD. In this study, the hub gene IGF1 is a member of growth factors, which can affect the development of IDD through various ways.

KEGG pathway analysis showed that signalling pathways regulating pluripotency of stem cells and p53 signalling pathway were closely related to IDD, and both were involved in IGF1. IGF1 has been implicated in age-related diseases and is a key factor in anti-cellular aging, extending the lifespan of species. In vitro studies have reported that IGF1 can promote the synthesis of extracellular matrix. Further in vivo studies have found that the reduction of IGF1 bioavailability can reduce the senescence of intervertebral disc cells and the decomposition of extracellular matrix [14], and promote the synthesis of proteoglycan and type II collagen in intervertebral disc cells [15], which was consistent with the results of IDD development caused by the down-regulation of IGF1 in this study. As the largest avascular tissue in the whole body, the nutrient supply of intervertebral disc mainly comes from the infiltration of cartilage endplate, and IGF1 helps to increase the shuttle of nutrients. Kusuma found that human wharton's jelly mesenchymal stem cells induced by IGF1 can repair damaged articular cartilage by reducing inflammation and inhibiting MMP3 [16]. For the p53 signalling pathway, Kim found that senescent nucleus pulposus chondrocytes increased with age and promoted the development of IDD through the telomere-based p53-p21-pRB pathway [17]. At present, the role of IGF1 in the p53 signalling pathway is mainly focused on tumours. IGF1 regulates tumour development by affecting cell proliferation, apoptosis, and senescence through the p53 signalling pathway [18, 19]. The pathway was also involved in the hub genes CCNB1 and CHEK1. CHEK1 was also used in zhang's study as a hub gene and new biomarker for IDD [20], a serine/threonine-specific protein kinase that regulates the DNA damage response and cell cycle checkpoint response and influences tumour development through both of these [21]. The regulatory relationship between CHEK1 and p53 in apoptosis affects the development of various diseases [22, 23]. CCNB1 has also been found to be involved in the development of IDD by regulating the proliferation and apoptosis of nucleus pulposus cells [24], and it can also affect the cell cycle by silencing the p53 signalling pathway to induce tumour cells senescence and apoptosis [25].

The role of hsa-miR-508-5p upstream of IGF1, CHEK1 and CCNB1 in IDD remains to be explored, but it has been extensively studied in various tumour tissues and cardiovascular [26, 27]. Hsa_circ_0074817 can competitively sponge hsa-miR-508-5p to act as ceRNA, correcting the negative regulation of IGF1/CHEK1/CCNB1 by hsa-miR-508-5p. The upstream hsa-miR-4741 of BIRC5, OIP5 and AR has been reported as a core in ceRNA of IDD [28], but this regulation axis still needs to be experimentally verified for reliability and validity. This study suggests that hsa_circ_0003600-hsa-miR-4741-BIRC5/OIP5/AR regulatory axis may also be one of the potential mechanisms of IDD, but there is a lack of research support, as does the hsa_circ_0002702-hsa-miR-3158-5p-IGF1/AR regulatory axis, which are the direction and goal of our future research.

Inflammation runs through the development of IDD, and immune cells are the main cause of inflammation. This study showed that IDD was also inseparable from the infiltration of immune cells. The expression of T cells CD8 was significantly decreased in IDD, while the expression of neutrophils was significantly increased. Compared with healthy people, the percentage of CD8 + T cells in patients with lumbar degenerative diseases was significantly lower, and the percentage of CD4 + T cells was significantly increased, which may be related to the changes in lymphocyte subsets and increased apoptosis of CD8 + T cells after IDD immune response. Neutrophils make up 40%-70% of all white blood cells in humans and are an important part of the innate immune system, which can reach and concentrate on the degenerated intervertebral disc through blood vessels, and release a large number of inflammatory mediators to destroy the microenvironment of the intervertebral disc, thus promoting the development of IDD [29]. We also found that the expression level of certain hub genes also affected the infiltration of immune cells. CHEK1 inhibition can augment CD8 + T cells infiltration in vivo models of multiple immunocompetent small cell lung cancer [30]. CCNB1 and BIRC5 have been reported to be related to the immune infiltration of hepatocellular carcinoma, and their expressions are positively correlated with the infiltration levels of CD4 + T cells and dendritic cells, respectively [31, 32]. OIP5 is one of the ideal targets for tumour immunotherapy, and the amount of its expression determines the expression of apoptosis-related genes, which affects cancer progression [33]. In addition to inflammation, apoptotic Fas protein and its ligand FasL mediating apoptosis of Fas positive cells play an important role in intervertebral disc immune amnesty. Studies have shown that FasL expression is significantly reduced in degenerating discs, and that FasL and its potential immune privileging mechanism retard disc degeneration by preventing infiltration of host immune cells [34]. The hub genes IGF1, CHEK1, CCNB1, BIRC5 and OIP5 play a role in anti-senescence and apoptosis, whether they can reduce the senescence and apoptosis of FasL to improve the intervertebral disc microenvironment needs further study. Bone marrow mesenchymal stem cells have great potential and hope in the treatment of IDD because of their low immunogenicity and immune regulation. IGF1 can greatly enhance the bioactivity of human-derived mesenchymal stem cells and activate the PI3K/Akt signalling pathway to play a protective role against hypoxia and nutrient deficiency, thus providing guarantee for mesenchymal stem cells to treat IDD [35]. Thus, circRNAs, miRNAs and mRNAs not only directly affect IDD, but also by regulating immune cell infiltration.

There are some limitations in this study. First, the sample size of the datasets included in the study was small and there were some missing samples when performing immune analysis, which may have an impact on the accuracy and reliability of the results, and a larger sample size is needed to support it. Second, although some of the key genes of the present findings have been reported in previous studies, the same lack of experimental and clinical validation is a direction for further research.

## Conclusion

In this study, we constructed and analysed the circRNA-miRNA-mRNA network by bioinformatics and found that the network not only mediates IDD by regulating cell senescence and apoptosis, but also affects IDD by influencing immune cell infiltration. These new findings will help us gain further insight into the pathogenesis of IDD and determine potential therapeutic targets.

## Data Availability

The datasets used and/or analysed during the current study are available from the corresponding author on reasonable request.
